# Visualization data on the freezing process of micrometer-scaled aqueous citric acid drops

**DOI:** 10.1016/j.dib.2016.11.037

**Published:** 2016-11-30

**Authors:** Anatoli Bogdan, Mario J. Molina, Heikki Tenhu

**Affiliations:** a Laboratory of Polymer Chemistry, Department of Chemistry, University of Helsinki, P.O. Box 55, FI-00014 Finland; b Department of Physics, University of Helsinki, P.O. Box 48, FI-00014, Finland; c Department of Chemistry and Biochemistry, University of California, La Jolla, San Diego, California 92093-0356, USA

**Keywords:** Emulsified aqueous citric acid, Freezing, Freeze-induce phase separation (FIPS), Freeze-concentrated solution (FCS), Lyophilization/freeze-drying

## Abstract

The visualization data (8 movies) presented in this article are related to the research article entitled “Freezing and glass transitions upon cooling and warming and ice/freeze-concentration-solution morphology of emulsified aqueous citric acid” (A. Bogdan, M.J. Molina, H. Tenhu, 2016) [1]. The movies recorded *in-situ* with optical cryo-miscroscopy (OC-M) demonstrate for the first time freezing processes that occur during the cooling and subsequent warming of emulsified micrometer-scaled aqueous citric acid (CA) drops. The movies are made publicly available to enable critical or extended analyzes.

**Specifications Table**

**Table:** 

Subject area	Pharmaceutics, Biotechnology, Tissue Engineering
More specific subject area	Freezing step in lyophilization
Type of data	Movies
How data was acquired	Movies were recorded using an Olympus BX51 optical cryo-microscope equipped with a Linkam cold stage, Linksys32 temperature control and video capture software.
Data format	Raw, processed
Experimental factors	Emulsified micrometre-scaled CA/H_2_O drops were prepared by magnetic stirring of CA/H_2_O solutions with a Halocarbon-0.8-oil/lanolin matrix.
Experimental features	Emulsion samples were placed in between a standard 75×25 mm microscope slide and a cover glass. OC-M measurements were performed at the cooling and warming rate of 3 and 5 K/min between 193 and 300 K.
Data source location	University of Helsinki, Finland
Data accessibility	Movies are presented in this article.

**Value of the data**

• The movies provide visual insights into the physical chemistry of freezing dispersed aqueous solutions and can be used by other researches who work with freezing phenomenon in fields ranging from life sciences and biotechnology to geophysics and high-altitude ice clouds.
• The movies demonstrating the freezing process were recorded *in-situ* using OC-M and can be compared to freezing results obtained with other techniques, for example, confocal fluorescence microscopy as well as to results obtained by computer simulations.
• Since the movies are first of their kind, they can be used/give an impetus in/for the development of further experiments in different fields of science and technology where freezing phenomena play important role.

## 1 Data

The movies presented in this data article provide the visualization evidence of a freeze-induced phase separation (FIPS) into pure ice and a freeze-concentrated solution (FCS) which occurs during the freezing of micrometer-scaled CA/H_2_O drops. These movies also demonstrate how the ice/FCS morphology of frozen drops changes with decreasing drop size.

## 2 Experimental design, materials and methods

We prepared 10–60 wt % CA solutions by mixing >99% anhydrous citric acid (Merck) with the corresponding amount of ultrapure water. For emulsion preparation we used an oil-surfactant matrix consisted of 80 wt % halocarbon 0.8 oil (Halocarbon Products Corp.) and 20 wt % lanolin (Sigma Aldrich). CA/H_2_O/oil-surfactant-matrix of 1/10 by volume were subjected to magnetic stirring at different speeds in order to obtain CA/H_2_O drops of different size distributions [1]. In our measurements, we used methodology based on a ´2-dimensionál´ solution strategy designed for the *in-situ* observation of FIPS and ice/FCS morphology by applying OC-M [2], [3].

## Appendix A Supplementary material

**Movie 1**. Freezing upon cooling of drops smaller than ~5–7 µm in diameter. The freezing process is seen as the subtle change of drop size and brightness.

.

**Movie 2**: Freezing upon cooling of drops smaller than ~100 µm in diameter. The freezing process is seen as the change of drop brightness and the appearance of channels issuing from freezing drops.

.

**Movie 3**: The resumed freezing of FCS and subsequent ice melting upon warming of frozen drops. The resumed freezing is seen as the increase of drop darkness. The melting of ice is seen as the appearance of quasi-spherical bulges that disappear as ice melts completely.

.

**Movie 4**: The freezing of concentrated drops from multiple ice nucleation events upon cooling. In the largest drop, there are 6 ice nucleation events.

.

**Movie 5**: Resumed freezing and subsequent melting upon warming of a large drop. The resumed freezing of FCS is seen as the growth of branched ice structure. A crystal of citric acid (CA) is seen on the left side of the drops.

.

**Movie 6**: Freezing upon cooling of 45 wt% CA drops smaller than ~100 µm in diameter. Some drops freeze from two and three ice nucleation events.

.

**Movie 7**: Resumed freezing and melting upon warming of 45 wt% CA drops which freezing upon cooling is shown in Movie 6.

.

**Movie 8**: Resumed freezing and melting upon warming of a ~400 µm drop. Movie demonstrates (i) the resumed freezing of FCS_2_, (ii) ice melting and (iii) dissolution of crystalline CA (see also the research article entitled “Freezing and glass transitions upon cooling and warming and ice/freeze-concentration-solution morphology of emulsified aqueous citric acid” (A. Bogdan, M.J. Molina, H. Tenhu, 2016) [1]).
